# Pressure Injury Link to Entropy of Abdominal Temperature

**DOI:** 10.3390/e24081127

**Published:** 2022-08-15

**Authors:** Nikhil Padhye, Denise Rios, Vaunette Fay, Sandra K. Hanneman

**Affiliations:** Cizik School of Nursing, The University of Texas Health Science Center at Houston, Houston, TX 77030, USA

**Keywords:** refined multiscale entropy, sample entropy, bubble entropy, complex adaptive system, pressure ulcer, machine learning, body temperature

## Abstract

This study examined the association between pressure injuries and complexity of abdominal temperature measured in residents of a nursing facility. The temperature served as a proxy measure for skin thermoregulation. Refined multiscale sample entropy and bubble entropy were used to measure the irregularity of the temperature time series measured over two days at 1-min intervals. Robust summary measures were derived for the multiscale entropies and used in predictive models for pressure injuries that were built with adaptive lasso regression and neural networks. Both types of entropies were lower in the group of participants with pressure injuries (n=11) relative to the group of non-injured participants (n=15). This was generally true at the longer temporal scales, with the effect peaking at scale τ=22 min for sample entropy and τ=23 min for bubble entropy. Predictive models for pressure injury on the basis of refined multiscale sample entropy and bubble entropy yielded 96% accuracy, outperforming predictions based on any single measure of entropy. Combining entropy measures with a widely used risk assessment score led to the best prediction accuracy. Complexity of the abdominal temperature series could therefore serve as an indicator of risk of pressure injury.

## 1. Introduction

Pressure injuries, or pressure ulcers, are caused primarily by extended exposure to pressure [1] and modified by the tissue’s tolerance to pressure [2]. Sustained high pressure can lead to decreased blood flow, occlusion of blood vessels and lymphatic vessels, and tissue ischemia [3]. In the conceptual model developed by Braden and Bergstrom [2], a tissue’s tolerance to pressure is affected by both extrinsic and intrinsic factors. Extrinsic factors include moisture, friction, and shear. Intrinsic factors include undernutrition; decreased arteriolar pressure; and other hypothetical factors such as interstitial fluid flow, emotional stress, smoking, and skin temperature. The etiology of pressure injuries continues to be an active area of research (e.g., [4]) complemented by advances in understanding the biomechanics of aging and wound healing [5] and in the role of skin microclimate [6].

Variations in skin temperature have been used in laboratory and clinical studies as a measure of the perfusion in the papillary dermis [7,8,9]. Methods for measuring microvascular blood flow include laser Doppler flowmetry [10], optical coherence tomography (e.g., [11,12]), and thermal infrared imaging (e.g., [13]). In the context of thermal infrared imaging research, it has been reported that it is possible to measure the effect of local blood circulation on skin temperature [14]. Peripheral vasoconstriction conserves heat by preventing heat loss from convection and radiation at the skin surface, whereas vasodilation increases blood flow and heat flow from the core to the epidermis [15]. Assuming that peripheral vasodilation and vasoconstriction behave like other organ systems under autonomic control, skin temperature under usual conditions should be highly irregular; i.e., variations in the skin temperature should be complex. Skin temperature can thus play a role as a proxy measure of the thermoregulatory function.

In the scientific framework of complex adaptive systems, the complexity of physiological variables arises from continuous adjustments to stimuli in order to maintain stability, and high complexity is a sign of youth and good health [16,17,18]. Measuring the complexity via entropy measures of skin temperature is thus a reflection of thermoregulatory properties of the skin, offering one way to quantify skin function. Skin failure has been implicated in propensity for pressure injuries [19], and the speed of skin temperature recovery following the relief of pressure from an externally applied indenter has been found to predict risk of pressure injuries [20,21]. Liao et al. [22] studied the complexity of blood flow oscillations with multiscale entropy [23] under a set of experimental conditions. Local heating and cooling of the skin were found to have distinct multiscale entropy signatures in the phase of reactive hyperemia that followed the release of pressure on the skin tissue. A word about the terminology surrounding complexity is in order here, because there are two main definitions of complexity [24]. *Algorithmic complexity* equates complexity to randomness or irregularity, which is the sense in which we use the term in this article. In contrast, *self-generated complexity* links the concept of complexity to the generation of meaningful structures.

In a previous study, Rapp et al. [25] broke new ground in reporting the association found between decreased multiscale entropy of abdominal skin temperature and the risk of pressure injuries. The main limitation of the study was that only three participants developed pressure injuries during the follow-up period. In another study, secondary analysis of data collected during a multinational randomized controlled trial [26] revealed that the fractal dimension of physical activity, a measure of its complexity, was a distinguishing factor between facility residents with pressure injuries and controls who were matched on the basis of several risk factors [27].

Despite advances in mattress technologies in recent years that have improved the pressure distribution in bed, incidence of pressure injuries has remained a concern in the US and elsewhere [26,28]. North American and European estimates of the associated economic burden are high: the median treatment cost is in excess of ten thousand dollars per incidence [29,30,31]. The Braden scale [32] is an efficient, well-studied, and widely used survey instrument to assess the risk of pressure injuries in hospitals and nursing facilities. However, it is not perfect, and the development of new approaches to improve assessment of the risk of pressure injury is an important goal that aligns well with the broader goals of personalized healthcare. Improved risk assessment offers the potential to provide targeted assignment of limited staffing resources in nursing facilities, and reduces the incidence of pressure injuries, along with the high cost of treating them.

The primary purpose of this study was to examine the association between pressure injuries and multiscale entropy of abdominal temperature. A guiding heuristic principle in the science of complex biological systems is that a state of reduced disorder is associated with disease or frailty from aging. Aligned with this postulate, the study hypothesis was that the incidence of pressure injuries would be elevated in participants with lower levels of multiscale entropy. Apart from contributing new, stronger evidence for a previously tentative finding [25], this study adds several innovations. Refined multiscale entropy [33] was used to improve some deficiencies of multiscale entropy. Bubble entropy [34] was added as a complement to sample entropy [35] because the two entropies differ sharply in their approach to evaluating the disorderliness of a time series. Robust summary measures of refined multiscale entropy were subsequently developed to reduce the dimensionality, and machine learning methods were used to predict pressure injuries on the basis of entropy values.

## 2. Methods

### 2.1. Study Design and Data Collection

The design was a prospective cohort study with time series measurements made over 48 h after participant enrollment. Skin was examined at baseline and weekly thereafter to detect occurrence and stage of pressure injury, over a period of three weeks. Residents were recruited from an urban nursing facility with 50-bed capacity. Informed consent was required before enrollment in the study, in accordance with procedures approved by the Committee for Protection of Human Subjects at The University of Texas Health Science Center and policies of the nursing facility. The recruitment target was set at n=40 based on consideration of power and the prevalence of pressure injuries. Using Poisson modeling based on the preliminary study [25], it was estimated that the target would yield an >0.5 chance of observing 6 or more pressure injuries in the study. Assuming the least prevalence in that range, along with the standardized effect size, d=1.13, estimated from the preceding study, power was found to exceed 0.80. The power was calculated in G*Power 3.1 [36] for a one-sided *t*-test design with α=0.05.

The study was impacted by the COVID-19 pandemic, and it was terminated after a futile wait of >18 months for the nursing facility to reopen for research activities. At the time of termination, n=28 participants had been recruited. However, the goal of 6 or more participants been found with pressure injuries was easily exceeded, and there were 12 such participants in the study sample.

Residents of the nursing facility who were age 70 or older were eligible to participate in the study. Eligible participants either had a pressure injury or were at risk of developing a pressure injury, indicated by Braden scale score ≤16. Other eligibility criteria included the ability to understand and provide informed consent. Exclusion criteria included an active infection indicated by body temperature elevated above 99.5 ${}^{\circ }$F.

Those who were at risk but did not develop a pressure injury during the study period will be referenced as *control* cases from here on, and the others will be referred to as *pressure injury* cases. Note that unlike common usage, *control* does not refer to healthy controls in this study. The *control* group here could be considered an *at-risk control* group.

Initial assessment of a new study participant included a skin examination and assessment of the pressure injury risk with the Braden scale. The temperature monitoring device was taped to the abdomen using water-resistant, hypoallergenic medical tape, approximately three inches to the left or to the right of the navel. The monitoring device was removed approximately 48 h later, and temperature data were downloaded to a secure server. Age, sex, and race of the participant; medications; number of comorbidities; dementia; and vascular conditions were noted by the Research Nurse, and vital signs were measured. Occurrence of pressure injury was monitored over the study’s duration in control cases, and injury stage was monitored in pressure injury cases. Data were maintained in a secure *REDCap* database.

### 2.2. Primary Measures

Skin temperature was measured with *iButton* high-density temperature loggers (Maxim Integrated, San Jose, CA, USA). The model *DS1922L iButton* has accuracy of ±0.5 ${}^{\circ }$C in the range −40 to +85 ${}^{\circ }$C. More importantly for the entropy estimation, it has 11-bit resolution of 0.0625 ${}^{\circ }$C, and sufficient memory for 4096 logged values. At 1-min intervals, the *iButton* thermochron could log temperatures for up to 2.84 days. The *iButton* is approximately dime-sized, weighs only 3.3 grams, and was well-tolerated by participants for the 48-h measurement duration.

The Braden scale [32] for measuring pressure injury risk is composed of 6 subscales: sensory perception, activity, mobility, nutrition, moisture, friction/shear. The subscales are scored 1 to 4, except for the friction/shear subscale, which is scored 1 to 3. The range of the total score is 6 to 23. The Braden scale differentiates risk categories based on the total score, ranging from very high risk below 10 to no risk above 18. The scale has widespread use, and interrater reliability has been reported to be high. There is also satisfactory evidence of validity and reliability [37].

Head-to-toe skin assessments were made by the study research nurse using established criteria [38]. A stage 1 pressure injury involves a persistent, nonblanchable erythema over a bony prominence in a light-skinned individual or red, blue, or purple hues in dark skin present at the same site on two consecutive days. A stage 2 injury has breaks in the skin, such as blisters or abrasions; a stage 3 injury has exposed subcutaneous tissue; and a stage 4 injury has exposure that extends into muscle or bone. Interrater reliability between the study Clinical Consultant and Research Nurse was assessed prior to study commencement; the mean was 0.87(range:0.85,0.90).

### 2.3. Entropy Measures

Sample entropy [35] is a widely used measure of entropy for time series data. Stable estimates can be produced on relatively short series lengths >10${}^{m}$, where *m* is the embedding dimension. Sample entropy (SampEn) is the negative natural logarithm of the conditional probability that epochs of length *m* that match point-wise within a tolerance *r* also match at the next point. Higher values of SampEn indicate smaller likelihood of continued matching of a pattern of size *m* at the (m+1)th point.

Multiscale entropy [23] was proposed to extend and improve the application of SampEn at temporal scales longer than the scale set by the sampling interval of the time series. The simple averaging used for coarse-graining in multiscale entropy is known to have poor properties as a low-pass filter. *Refined multiscale entropy* [33] uses a Butterworth filter to improve the elimination of fast temporal scales, providing a flat response in the passband, with fast roll-off and elimination of side lobes in the stopband. Refined multiscale entropy additionally counteracts artificial shrinking of entropy at longer scales by updating the tolerance *r* as a percentage of the standard deviation of the filtered series rather than that of the original series.

In this study, refined multiscale SampEn was estimated for each abdominal temperature series using the *EntropyHub* library [39] that has been developed for use with multiple programming languages, including *Python*, which was used here. A sixth-order Butterworth filter with cutoff frequency 1/2τ for timescale τ was used for low-pass filtering of the time series. Although the original introduction of multiscale entropy involved the calculation of SampEn at each scale, *EntropyHub* has made it easy to expand and apply the notion of multiscale entropy to other types of entropy measures that were introduced later [40].

Bubble entropy [34] evolved from permutation entropy [41], which is based on the number of steps needed to sort the embedded sequence in ascending or descending order. This approach sets it apart from the tolerance based pattern matching approach of SampEn. Therefore, we supplemented the entropy measures with the refined multiscale bubble entropy. A similar approach was adopted in a study that used sample entropy and permutation entropy to improve classification of fever from body temperature signals [42].

Bubble entropy (BubbEn) is calculated from the conditional Rényi entropy of the probability distribution of number of swaps needed to sort the embedded sequences using the well known *bubble sort* algorithm. BubbEn at embedding dimension *m* is a normalized difference of the conditional Rényi entropies at *m* and m+1 dimensions. Like SampEn, BubbEn has been shown to converge at short series lengths. Moreover, BubbEn has only one parameter, *m*, and estimated values have less sensitivity to the choice of *m* than other entropies [34].

Time series lengths were sufficient to investigate SampEn for embedding dimensions m=2,3. The tolerance parameter for SampEn was investigated for values r=0.10,0.15,0.20, which represent fractions of the standard deviation of the time series for pattern matching. BubbEn was calculated for embeddings m=2to10. The longest temporal scale for investigation of refined multiscale entropies was set to τ=25 min so that the time series lengths were >100 times the maximum scale, on average.

### 2.4. Temperature Time Series

**Figure 1:**
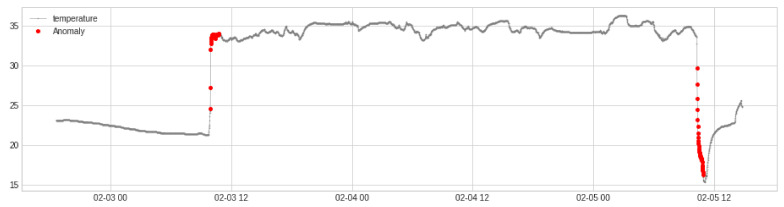
Temperature time series data are shown over a period of 68 h, along with detected autoregressive anomalies that aided in identification of the active section with abdominal temperature measurements.

The *iButton* temperature sensor was in continuous logging mode after a software reset that was typically carried out a few hours before data collection began. Times of mounting and removing the temperature sensor on or off the participant’s abdomen were noted as part of the protocol. Sensor *on* and *off* times were further fine tuned by tracking changes in the pattern of autoregressive behavior of the time series, detected with the *ADTK* package [43] for *Python*, which was executed in the *Google Colaboratory* environment. See Figure 1 for an example that shows one cluster of anomalies near the *on* time and another cluster of anomalies near the *off* time. Sections of the time series between the rightmost anomaly in the *on* cluster and ending 30 min prior to the first anomaly of the *off* cluster were selected for further analysis with the entropy methods described in Section 2.3. Stationarity is required for assessment of entropy [44], which was assessed with the augmented Dickey–Fuller test for the unit root [45]. Linear detrending was sufficient to achieve stationarity in all but three cases, for which removal of the circadian rhythm with a simple cosine fit was used to minimize changes to the structure of the series.

The median length of the selected series was 2800 (46.7 h), and the interquartile range was 2630 (43.8 h) to 2826 (47.1 h). One series was of substantially shorter duration (24.0 h) than all others due to unexpected hospitalization of the participant for a reason unrelated to the study. Two temperature series were discarded due to errors in executing the software reset of loggers. Useful temperature data were available for 15 of 16 control cases and 11 of 12 pressure injury cases.

Summary measures of time series were calculated with the aim of studying any differences in distributions between pressure injury and control groups. The median was used as a measure of the central tendency of abdominal temperature, along with the interquartile range (IQR) as a measure of dispersion. The trimmed 95% range between the 2.5th and 97.5th percentiles was also examined as a simple proxy measure of the amplitude of circadian variation.

### 2.5. Data Analysis and Models

#### 2.5.1. Summary Measures of Multiscale Entropy

The refined multiscale entropy measures described in Section 2.3 resulted in a series of SampEn and BubbEn values at each scale per participant. One approach for shrinking the parameter space for statistical modeling would be to select the particular scales for each type of entropy that provide the highest power to discriminate between the injury groups. However, such a measure arising from a single scale may have a degree of stochasticity that could make it a feature of the sample. Aiming to find summary measures of multiscale entropy that are more generalizable, even if they might result in loss of statistical power, we focused on two properties that span a range of scales: shape of the entropy-scale curve, and a measure of the magnitude associated with the curve.

The simplest measure of the shape is the slope of the curve centered between any two specified scales. For example, in the Taylor series approximation of a function in calculus, an extension along the first derivative, or slope, serves as the first order approximation, followed by a contribution from the second derivative, which is related to curvature, and then on to smaller terms arising from higher-order derivatives. We define the *scaling exponent* as the slope of the entropy curve on the logarithmic scale:

(1) $$\mathrm{Scaling}\;\mathrm{Exponent}\left(s\right)={\left.\frac{d\;\mathrm{En}(\tau )}{d\ln(\tau )}\right|}_{s}\;$$

where En(τ) denotes either SampEn or BubbEn at scale τ. The slope is evaluated at scale *s*, which was selected to maximize the effect size of pressure injury, conditional upon spanning at least five consecutive scales. In practice, the scaling exponent is estimated by the parameter resulting from a regression of entropy on the logarithmic scale, which necessitates the selection of optimal limits $s_{1}$ and $s_{2}$ for the scale range. While these ranges could also vary under sampling, the inclusion of entropic structure across many scales makes this measure more robust than selecting a single scale.

Area under the curve (AUC) between any two specified scales is a natural measure of the magnitude of entropy that spans more than one scale. We define the *requisite AUC* measure by

(2) $$\mathrm{Requisite}\;\mathrm{AUC}\left(s_{1},s_{2}\right)=\int_{s_{1}}^{s_{2}}\mathrm{En}\left(\tau \right)\;d\tau \;$$

where En(τ) denotes either SampEn or BubbEn at scale τ. The limits of the integration range, $s_{1}$ and $s_{2}$, were selected by maximizing the effect size of pressure injury conditional upon spanning at least three consecutive scales to enhance robustness.

It may be worth noting that these summary measures can be calculated independently for each individual without needing to know weights or parameters that could depend on other individuals. For example, principal components can only be calculated on the entire collection of individuals, rendering them dependent on sampling. The scaling exponent and requisite AUC measures are independent of sampling, and they need only a single time series for their estimation. The analysis was scripted in the *R* programming language [46], and it was executed in an *RStudio* environment [47].

#### 2.5.2. Simple Bivariate Models

The differences in the means of the refined multiscale SampEn and BubbEn between pressure injury and control groups were assessed at each scale from the standardized effect size, *d*, and with two-sample Welch *t*-tests. Since this was an exploratory study rather than a confirmatory one, we did not control the family-wise error rate for multiple tests. The emphasis was on the effect sizes rather than *p*-values. Differences between groups of the scaling exponent and requisite AUC of multiscale SampEn and BubbEn, discussed in Section 2.5.1, were assessed in a similar manner.

Finding a difference in the mean of an entropy measure between pressure injury and control groups does not automatically guarantee that reversal of the dependent and independent variables will lead to a satisfactory model for predicting pressure injury from measurements of the entropy. Since any practical application of finding an appreciable difference in entropies would be geared toward prediction of the risk of pressure injury, we modeled it explicitly with machine learning methods that are described next.

#### 2.5.3. Predictive Models

Two types of predictive models for pressure injuries are presented. One type of model is more traditional in the sense that it provides inferences about the links between pressure injury and the predictors, and the other type is focused more on prediction accuracy than inference. Generalized regression uses shrinkage methods and incorporates validation sets in the model construction and evaluation process. Adaptive lasso regression with leave-one-out cross-validation was used for term selection [48]. Adaptive lasso regression is a type of generalized regression that penalizes regression coefficients based on their size, shrinking some of the coefficients to zero, trading bias for reduced variance in the estimates [49,50]. Lasso regression is immune to multicollinearity, and it is known to work well even when there are large number of predictors relative to the size of the dataset, making it suitable for this study with its limited sample size. A second lasso step was used to explore the second-order interaction terms of all terms that were left in the model after the first run. The second type of predictive model was a neural network, which was restricted to have only a single hidden layer and no more than two nodes to minimize the risk of overfitting [51]. Models were built with 5-fold cross-validation, and the selection of activation functions for the nodes is described in Section 3.3.2. Adaptive lasso regression and neural models were built and executed using *JMP Pro* (version 15.2).

## 3. Results

### 3.1. Sample Description

Distributions of demographic variables, history of comorbidities, body weight, vital signs, and Braden Scale score assessed at baseline are shown in Table 1. Descriptive statistics are displayed for the sample, in addition to being split by the pressure injury status. The control and pressure injury groups are defined in Section 2.1; briefly, control cases did not develop a pressure injury during the study period. The table includes the descriptions of summary measures of the time series temperature data. Among participants with pressure injuries, the maximum stage of injury observed during the study period was close to uniformly distributed across stages: n=3 for stages 1 and 2, n=4 for stage 3, and n=2 for stage 4. The location of injury was uniformly distributed with n=3 each for sacrum or coccyx, ischial tuberosity, heel, and other location.

### 3.2. Multiscale Entropy and Pressure Injuries

#### 3.2.1. Scale Structure of Entropies

**Figure 2:**
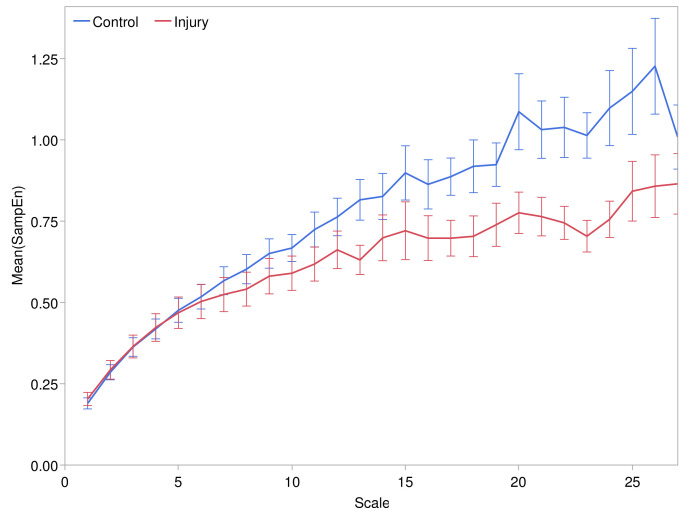
Refined multiscale sample entropy (m=3, r=0.15) at temporal scales ranging from 1 to 25 min. Error bars depict the standard error in the control and pressure injury groups.

Refined multiscale SampEn tended to increase over temporal scales from 1 to 25 min, as shown in Figure 2. The embedding dimension was m=3, and tolerance parameter r=0.15. The rate of increase was faster at the lower scales, particularly in the control group, and decreased at higher scales. The mean SampEn level tended to be lower in the pressure injury group relative to the control group at scales exceeding 7 min. The pattern was similar for embedding dimension m=2 and variations of ±0.05 in *r*, but m=3 and r=0.15 yielded an excellent distinction between the control and pressure injury groups.

**Figure 3:**
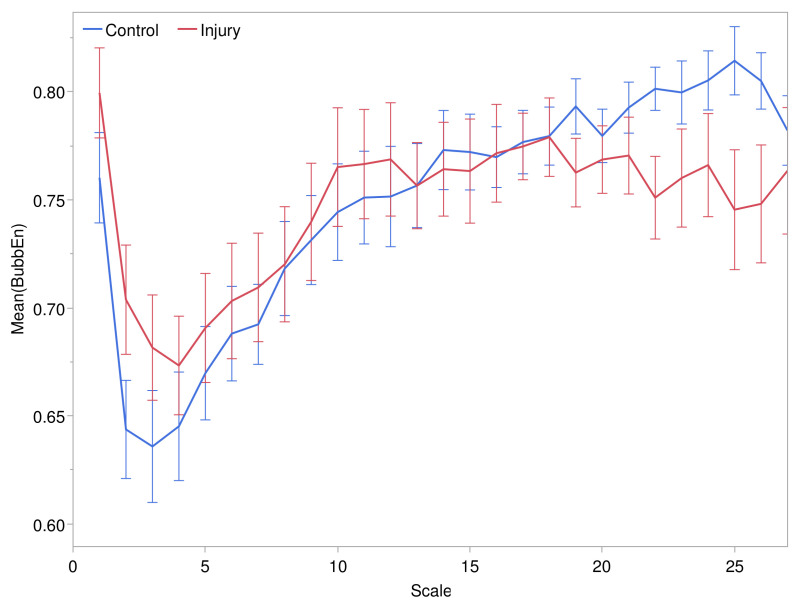
Refined multiscale bubble entropy (m=3) at temporal scales ranging from 1 to 25 min. Error bars depict the standard error in the control and pressure injury groups.

Refined multiscale BubbEn tended to increase over temporal scales from 4 to 25 min, as shown in Figure 3 for embedding dimension m=3. The rate of increase of BubbEn had a transition point near the 11-min scale, beyond which the mean entropy flattened out in the pressure injury group, whereas it continued to increase in the control group. The pattern was similar for embedding dimension m=2, but m=3 yielded excellent distinction between the control and pressure injury groups. BubbEn was also explored for m>3, being up to 10. At these higher values of *m*, the effect size of pressure injury appeared to be more stochastic across scales, whereas there was a more stable scale structure for m≤3.

#### 3.2.2. Comparison of Entropies by Pressure Injury Group

**Figure 4:**
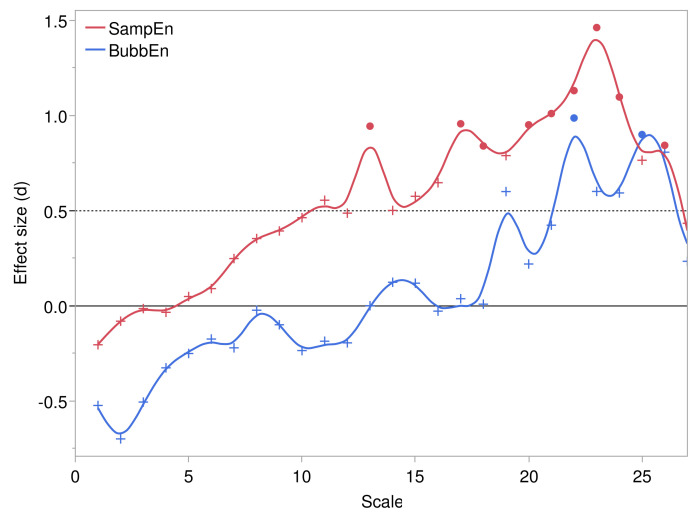
Effect sizes, i.e., standardized mean differences (Cohen’s *d*) between control and pressure injury groups, at each temporal scale for refined multiscale sample entropy and bubble entropy. Filled circles indicate effects that satisfied p<0.05.

Comparisons of the mean entropy levels at each scale between control and pressure injury groups showed a generally increasing effect size over scales, peaking at 22 and 23 min for SampEn and BubbEn, respectively. Cohen’s *d* effect size, defined by the difference in means measured in standard deviation units, is shown against scale in Figure 4. Due to the limitation of sample size, only large effects, d>0.9, were statistically significant in inference with the independent samples *t*-test. Nevertheless, the extent of consecutive scales with d>0.5 suggest robustness of the pressure injury effect on entropy levels.

The scaling exponent and requisite AUC measures that summarize the refined multiscale SampEn and BubbEn curves for a participant were calculated as outlined in Section 2.5.1. The scaling exponent for the SampEn curve was derived by regressing entropy for a given participant on centered scales spanning from 5 to 10 min, and the scaling exponent for BubbEn spanned from 1 to 25 min. The specified scale ranges were obtained by a systematic search based on optimizing the effect size due to pressure injury. The use of centering on the logarithmic scale implies that the scaling exponents represent the parameter values at the centers of the ranges, which were located at 7.1 min for SampEn and 5.0 min for BubbEn. The equisite AUC spanned the range between 22 and 24 min for SampEn and 21 to 23 min for BubbEn, which includes the scale with peak effect for either type of entropy. The comparison of these summary measures between injury groups is shown in Table 2. The table includes the peak effect at the single scale. An unexpected bonus was that the scaling exponent for SampEn displayed a larger pressure injury effect than the best single scale, thereby providing a desirable combination that captured the scaling structure of the multiscale entropy along with increased statistical power.

### 3.3. Prediction of Pressure Injuries

#### 3.3.1. Generalized Regression Models

Pressure injury outcome was predicted with a series of generalized regression models ranging from simple bivariate models to fully adjusted models. Adaptive lasso regression was used for all models with incorporation of leave-one-out cross-validation. This resulted in generally improved performance over logistic regression, particularly for the multivariate models. Table 3 summarizes bivariate models that predicted pressure injury from each of four summary measures of refined multiscale entropies and the Braden scale score, considered one at a time. The model performance, assessed by area under the receiver operating characteristic (ROC) curve, generally tracked the order of effect sizes displayed earlier in Table 2.

Model *B2* serves as a reference with the Braden scale score as the sole predictor. The area under the ROC curve was 0.740, and accuracy for predicting controls was 94.1%, whereas only 58.3% of pressure injuries were correctly predicted with the classification threshold set at probability >0.5. Changing the classification threshold to 0.4 switched the imbalance to the opposite end: accuracy was 75.0% for pressure injuries, but only 47.1% for controls.

In contrast, model *B5*, based on predictions from the SampEn scaling exponent, had better balance and smoother changes in accuracy upon altering the classification threshold. When pressure injuries were predicted by probability >0.5, accuracy was 80.0% for controls and 90.9% for correct prediction of pressure injury cases. However, we note that predictions on the basis of individual entropy measures are capped by area under the ROC curve of 0.86, which leaves some room for improvement with multivariate models.

Next, we present and evaluate multivariate models that incorporated the summary measures of entropies and controlled for covariates. The models are summarized in Table 4. Model *M1* was the culmination of a process that started with inclusion of all summary measures of entropies. The adaptive lasso regression with leave-one-out cross-validation did not eliminate any of the four entropy measures from the set of predictors. Interaction terms were explored in the second step and found to be unnecessary. Model performance improved relative to the bivariate models, as indicated by the area under the ROC curve, 0.940. When pressure injury cases were classified with a threshold, probability >0.5, the accuracy was 80.0% for prediction of injuries and 86.7% for non-injuries. The overall misclassification rate was 16.0%. Decreasing the classification threshold to 0.30 yielded 100% accuracy for pressure injury cases, and the overall misclassification rate stayed at 16.0%.

Model *M2* resulted from a procedure that was identical to that of model *M1*, except that it included any covariates measured at the baseline that were potentially different in the groups. Covariates included the Braden scale score, sex, BMI, dementia, vascular disease, heart rate, and the median and trimmed range of the temperature time series. The Braden scale score was the only covariate that was retained along with the entropy measures that are listed in Table 4. Second-order interaction terms were found to be unnecessary. The model’s performance was very good, as indicated by the area under the ROC curve of 0.94. When pressure injury cases were classified with a classification threshold of 0.5, the accuracy was 100% for prediction of non-injuries, and the overall misclassification rate was 8.0%.

#### 3.3.2. Neural Models

Neural models provided improved classification accuracy over the generalized regression models. Neural networks were restricted to a single hidden layer, and 5-fold cross-validation was used in model development to mitigate the risk of overfitting. Since the sample size was small, further precaution was taken to restrict the number of predictors to only the most effective ones that were indicated by the generalized regression models discussed in Section 3.3.1.

**Figure 5:**
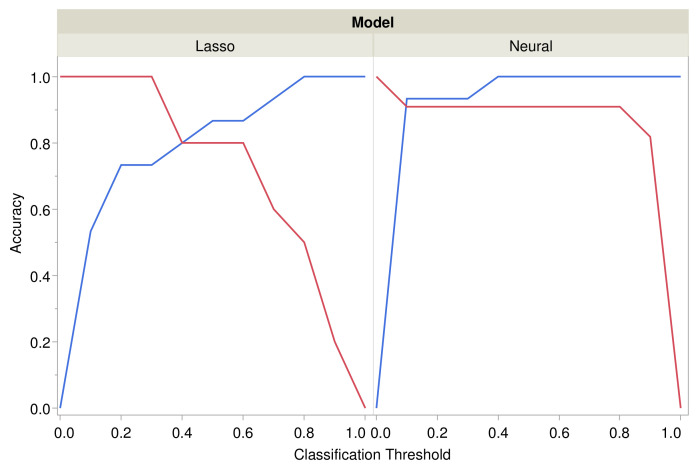
Accuracy of classification by adaptive lasso (model *M*1) and neural network (model *N*1) for prediction of pressure injury from the SampEn scaling exponent and BubbEn requisite AUC. Accuracy is shown separately for predicting pressure injury cases (red) and control cases (blue).

In the first neural model *N*1, pressure injuries were predicted from SampEn and BubbEn scaling exponents. The model had two nodes with a hyperbolic tangent activation function for one node and a Gaussian function for the other node. The area under the ROC curve was 0.946, and the classification accuracy was 100% for controls and 90.9% for pressure injuries; the overall misclassification rate was 4.0%. The corresponding threshold for classification was 0.5. Comparison of the accuracy of the adaptive lasso regression model *M*1 and neural model *N*1 is shown in Figure 5.

Addition of the Braden scale score as the third predictor in model *N*2 resulted in a perfect ROC area measure of 1.0 with 100% accuracy for predicting controls and pressure injuries. This model had a single layer with two nodes with a hyperbolic tangent activation function for one node and a Gaussian function for the other node. For network diagrams, parameter estimates, and other information, see Appendix A and Supplementary Material.

## 4. Discussion

A primary motivating factor behind the introduction of multiscale entropy was to reconcile apparent violations of a basic premise of the science of complex adaptive systems: higher complexity is generally indicative of a healthier system [23]. Although it may be violated at some scales, the basic truth behind this premise is made evident only when entropy is measured at temporal scales other than the one set by the choice of sampling frequency. For example, atrial fibrillation can lead to high entropy in the beat-to-beat series at short timescales but not at longer timescales. In this study, we found similar behavior for the refined multiscale SampEn and BubbEn of abdominal skin temperature. Participants without pressure injuries tended to have higher levels of entropy than participants with pressure injuries, but this was generally true only at temporal scales that were several times larger than the 1-min sampling period.

The differences in the mean entropy levels at the longer scales had large effect sizes due to pressure injury, consistent with the only other study on this topic that reported an effect size of roughly the same magnitude, d>1, for multiscale SampEn of abdominal skin temperature [25]. That study included only three participants with pressure injuries, focused on a single measure of entropy, and included participants at low risk of pressure injuries. The new measurements of complexity in eleven participants with pressure injuries contribute stronger evidence that lower levels of SampEn and BubbEn are associated with the injury state. Moreover, this association was observed despite the exclusion of low-risk participants in the present study.

The need to maximize the observations of relatively few participants with pressure injuries was an important factor in the study design. Had the study been limited to observations of new pressure injuries in the three-week follow-up time, it would have been far too restrictive. Allowing participants to enter the study with a pre-existing pressure injury provided valuable data on participants with pressure injuries, at the expense of losing the ability to examine the causal structure. Therefore, the present study cannot conclude that loss of entropy precedes the development of pressure injuries. However, the previous study by Rapp et al. [25] had an exclusively longitudinal design, and its findings suggest that low entropy preceded the development of pressure injuries by a few days to a few weeks. A more resource-intensive study will be necessary to follow large numbers of participants over longer periods of time to make firmer judgments about causality.

The addition of refined multiscale BubbEn was found to be a useful complement to the refined multiscale SampEn. Large effects were observed for the differences in means between groups of the SampEn scaling exponent (d=1.53), SampEn requisite AUC (d=1.38), and BubbEn scaling exponent (d=1.04). Despite having a smaller effect size than SampEn on a bivariate basis (see Table 2), in multivariate regression models it was the scaling exponent of BubbEn that edged out the scaling exponent of SampEn as the best predictor of pressure injuries (see Table 4). These two predictors can be thought of roughly as measures of the shape of the multiscale SampEn curve and the multiscale BubbEn curve, with the caveat of being restricted to certain scales that are discussed in Section 2.5.1. The neural models further confirmed that the scaling exponents of SampEn and BubbEn performed well together as predictors of pressure injury, yielding an overall 96% accuracy for the prediction of pressure injury and non-injury cases. This level of accuracy was unmatched by any single measure, regardless of whether it arose from SampEn or BubbEn.

Use of machine learning methods to predict pressure injuries on the basis of entropy measures resulted in models that performed well, with areas under the ROC curve not less than 0.94 for generalized regression with the adaptive lasso method and for the neural model. These models were superior to predicting pressure injuries on the basis of Braden scale scores alone. The predictors that were rejected by generalized regression are also noteworthy. Summary measures of abdominal temperature, including measures of variation such as the interquartile range and the trimmed range, did not differ between pressure injury and control groups. Entropy measures derived from the time series data were therefore crucial to detecting the pressure injury effect that could not be detected with simple measures of dispersion.

However, the best predictive models resulted from combining the entropy measures and the Braden scale score. Areas under the ROC curve for these models exceeded 0.96 for the generalized regression with the adaptive lasso method and for the neural network. A major advantage of the Braden scale is that it is in widespread use in nursing facilities. Therefore, combining this widely used score with experimental entropy measures derived from abdominal skin temperature might be the most straightforward and sensible next step to judge the risk of pressure injuries for residents of nursing facilities.

Nevertheless, it must be noted that the study design was such that it could introduce bias in the Braden scale score. The scoring was done by the research nurse on many of the participants with the knowledge that they had pressure injuries, creating the possibility of implicit bias in the scoring. The entropy measures, on the other hand, were calculated from sensor measurements, which made them immune to any subjective bias.

Entropy measures are not without their disadvantages. Roughly 42 h of temperature series data collection may be needed to obtain good estimates of entropies up to the scale at 25 min, which was the approximate location of the largest effect sizes. Alternatively, to estimate the most effective summary measure of multiscale entropy found in this study, the scaling exponent of the SampEn curve between scales 5 and 10 min, roughly 17 h of data collection may be needed. Measurement of entropy, therefore, involves a much longer duration than scoring the Braden scale, making it a process that is unlikely to be tolerated by all patients or residents of a facility. This should also demand patience from the staff or healthcare providers to wait for risk assessment to be completed from the entropy measurement.

The wide variety of entropy metrics that have been developed [40] can be both an advantage and a challenge. In this study, we used sample entropy and bubble entropy advantageously; however, we cannot rule out the possibility that there could be a different combination of entropies that provides more power to predict pressure injuries. The susceptibility to noise of various entropy metrics is another challenge. For instance, it has been shown in the context of heart period data that permutation entropy, closely related to bubble entropy, is more susceptible to the introduction of broad band noise than coarse-grained entropy [52]. Further studies of the impact of noisy temperature measurement on entropy levels could be helpful for assessing conditions under which they can be reliable measures.

Despite some limitations that we noted, entropy measures hold promise as objectively measured predictors of the risk of pressure injuries. The proposed underlying mechanism is that the entropies provide an assessment of the orderliness of temperature fluctuations that are linked to changes in the blood flow in the skin tissue. The fluctuations that occur on the timescales of a few minutes to about thirty minutes appear to be of prime importance. In healthier skin, the blood flow is likely to be more adaptive and variable, responding more dynamically to changes in surface pressure and temperature. The corresponding entropy level is therefore likely to be higher than in a state of unhealthy blood flow and/or thermoregulation. The lower state of health of the skin thermoregulation, in turn, is likely to raise the probability of the person experiencing a pressure injury when their skin is subjected to external stress.

It is known from laser Doppler flowmetry that there are a few characteristic oscillations in human peripheral blood flow [53]. While these oscillations were found at fast timescales (<2 min) in relation to the timescales pertinent to the present study, it suggests that there could be more structure present at slower timescales. This structure could arise from an interplay between the autonomic nervous system and the vascular system, and mirrored in corresponding structures in the temperature signal. Evidence for the interplay comes from studies of baroreflex that sometimes include assessment of peripheral resistance that tends to be under sympathetic control [54] and studies of oscillations arising from such control (e.g., [55]). It is also known that vasomotion diminishes with advancing age, and persistent obstruction of blood flow through the microvasculature can lead to the formation of microthrombi, which further obstructs blood flow [56,57]. Inspection of the power spectral density calculated with the Welch method [58] indicated that there were some differences in the spectral distribution between the two groups. The pressure injury group had >40% more power at several timescales (inverse frequency) between 2.4 and 9.1 min. It may be noteworthy that this interval includes the central points of the scale range for calculation of the spectral exponents of SampEn and BubbEn. In contrast, the control group had >75% more power at several timescales between 17.1 and 102.9 min. Overall, the power spectral density suggests that there was a shift of power from slower to faster timescales in the injury group. Such a shift could result in higher periodicity at timescales that are faster (smaller) than 10 min, approximately. It is plausible that this could lead to reduction in the irregularity of abdominal temperature that is detected by lowered entropy. However, this interpretation about the shift in the frequency structure is necessarily speculative and only serves as a hypothesis for a future study that will ideally be done with concurrent measurements of blood flow.

It may be worth noting that our approach based on evaluation of the irregularity of abdominal skin temperature as a proxy measure of skin thermoregulation is not to be confused with approaches that monitor skin temperature localized to the most commonly anticipated wound locations, such as the sacrum. Relative differences in skin temperature between zones can give an early indication of a developing pressure injury [59,60,61,62]. Localized approaches can result in early detection of pressure injuries, but they require clinicians or nursing facility staff to frequently scan several suspect areas for thermal imaging, which can be labor intensive. The approach presented in this study of using abdominal temperature monitoring to assess system-wide status of skin thermoregulation holds the promise of a low impact on staff workload, which is important in a climate of global shortages of health care providers.

One avenue for future studies could focus on establishing the causal pathway through laboratory studies or numerical simulations of blood flow, tissue thermodynamics, and tissue mechanics. Another avenue is from the risk assessment perspective for which longitudinal studies with longer follow-up times are required. The sample size of such a study would need to be large to offset the relatively low occurrence of pressure injuries. If low entropy levels of abdominal temperature precede the development of pressure injuries, it would make a strong case for regular monitoring of residents in nursing facilities or elsewhere. In such a study, it would be desirable to include blood flow monitoring and measurements of peak pressure in common bed postures for the participants. This would allow studying the link between temperature and blood flow and yield a better understanding of the frequency structures of these signals. The combination of risk assessment studies and engineering studies could also point the way to a treatment that can be offered to normalize the blood flow during a phase of high risk of pressure injury that might have been identified from the skin temperature measurements. For example, electrical stimulation has been proposed to increase periwound skin blood flow for nonhealing pressure injuries [63], which suggests a possibility that it could be used more effectively during an identified high-risk period before the injury is manifested.

## Figures and Tables

**Table 1:** Distribution of participant demographics and covariates by pressure injury group.

Characteristic	Control ^1^	Pressure Injury ^1^	Total ^1^
	($n_{1}=16$)	($n_{2}=12$)	(n=28)
Age (y)	78 (66, 83)	74 (68, 82)	75 (67, 82)
Sex:			
Male	7 (44%)	8 (67%)	15 (54%)
Female	9 (56%)	4 (33%)	13 (46%)
Race:			
Black	8 (50%)	7 (58%)	15 (54%)
White	8 (50%)	5 (42%)	13 (46%)
Num. Comorbidities	8 (5, 10)	8 (6, 9)	8 (6, 9)
Unknown	0	2	2
Dementia	3 (19%)	1 (8%)	4 (14%)
Vascular Disease	12 (75%)	10 (83%)	22 (79%)
Treated Vasc. Dis.	12 (75%)	9 (75%)	21 (75%)
Heart Rate (bpm)	81 (68, 90)	69 (63, 81)	77 (66, 89)
Blood Pressure (mm-Hg)			
Diastole	75 (65, 81)	72 (60, 78)	73 (64, 80)
Systole	137 (124, 154)	132 (119, 141)	136 (122, 144)
Temperature (${}^{\circ }$F)	98.2 (97.9, 98.6)	97.6 (97.1, 98.1)	98.0 (97.5, 98.4)
BMI (kg/m${}^{2}$)	28.2 (25.1, 37.6)	25.7 (21.3, 32.1)	27.2 (24.5, 33.6)
Weight (lb)	184 (145, 225)	168 (141, 220)	169 (144, 222)
Braden Scale Score	15.5 (15, 16)	14 (13, 15.8)	15 (14, 16)
Time Series Summary			
Median (${}^{\circ }$C)	35.1 (34.4, 35.9)	35.1 (34.2, 35.9)	35.1 (34.3, 35.9)
Interquartile Range (${}^{\circ }$C)	1.1 (0.8, 1.3)	0.9 (0.8, 1.3)	1.0 (0.8, 1.3)
Trimmed Range (${}^{\circ }$C)	3.1 (2.3, 3.6)	3.0 (2.4, 3.9)	3.0 (2.4, 3.6)
Unknown	1	1	2

^1^ Median (interquartile range) or n (%).

**Table 2:** Mean differences in entropy measures between control and pressure injury groups. The scaling exponent and requisite AUC measures are described in Section 2.5.1 and Section 3.2.2.

Characteristic	Eff. Size	Difference	95% Conf. Int.		*p*
	*d*		Lower	Upper	
SampEn					
Single scale (τ=23)	1.46	0.310	0.134	0.486	0.001
Scaling exponent	1.53	1.113	0.522	1.704	<0.001
Requisite AUC	1.38	0.628	0.248	1.009	0.003
BubbEn					
Single scale (τ=22)	0.99	0.050	0.005	0.096	0.033
Scaling exponent	1.04	3.079	0.624	5.534	0.016
Requisite AUC	0.82	0.081	0.008	0.170	0.035

**Table 3:** Bivariate adaptive lasso regression models for pressure injury predicted separately by each of the entropy measures and by the Braden scale score. Models are arranged in ascending order of area under ROC curve.

Model	AUROC ^a^	Term	OR ^b^	95% Conf. Int. (OR)		*p*
				Lower	Upper	
B1	0.727	BubbEn req. AUC	6.3 × 10 ^−4^	2.9 × 10 ^−7^	1.4	0.061
B2	0.740	Braden Score	0.47	0.26	0.84	0.012
B3	0.782	BubbEn scaling exp.	0.70	0.55	0.88	0.003
B4	0.807	SampEn req. AUC	3.9 × 10 ^−2^	8.3 × 10 ^−3^	0.18	<0.001
B5	0.861	SampEn scaling exp.	0.15	0.05	0.42	<0.001

^a^ Area under ROC curve. ^b^ Odds ratio

**Table 4:** Multivariate adaptive lasso regression models for pressure injury predicted by entropy measures (model *M1*), or after adjusting for all covariates, including the Braden scale score (model *M2*).

Model	AUROC ^a^	Term	OR ^b^	95% Conf. Int. (OR)		*p*
				Lower	Upper	
M1	0.940	BubbEn scaling exp.	0.58	0.36	0.93	0.025
		SampEn scaling exp.	0.21	0.05	0.88	0.033
		SampEn req. AUC	0.19	3.7 × 10 ^−2^	1.02	0.053
		BubbEn req. AUC	1.6 × 10 ^−9^	1.2 × 10 ^−18^	2.05	0.058
M2	0.967	Braden Score	0.25	0.12	0.54	<0.001
		BubbEn scaling exp.	0.68	0.49	0.95	0.024
		SampEn scaling exp.	0.25	7.5 × 10 ^−2^	0.85	0.026
		BubbEn req. AUC	6.8 × 10 ^−2^	1.7 × 10 ^−7^	2.6 × 10${}^{4}$	0.682
		SampEn req. AUC	0.88	0.20	3.9	0.867

^a^ Area under ROC curve. ^b^ Odds ratio

**Figure A1:**
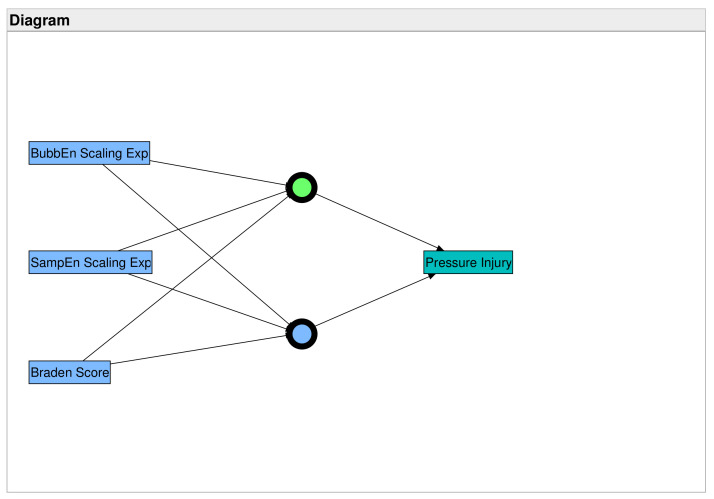
Structure of the neural network for prediction of pressure injuries from two entropy measures and the Braden scale score. The different colors of the nodes denote Gaussian and hyperbolic tangent activation functions.

## Data Availability

Deidentified data for multiscale entropy and predictive models presented in this study are openly available in Harvard Dataverse at https://doi.org/10.7910/DVN/CQ8FLL,referencenumberUNF:6:TsxIa1nvXmYCzrcDjCb4Wg== (accessed on 2 August 2022).

## Supplementary Materials

The following supporting information can be downloaded at: https://www.mdpi.com/article/10.3390/e24081127/s1, statistical details about the neural models.

## Abbreviations

The following abbreviations are used in this manuscript:

SampEn	Sample Entropy
BubbEn	Bubble Entropy
AUC	Area Under Curve
ROC	Receiver Operating Characteristic (Curve)
AUROC	Area Under ROC Curve
IQR	Interquartile range

## Appendix A

This section is in reference to the neural network models that were described in Section 3.3.2. An example of the neural network structure for model *N2* is shown in Figure A1. Please refer to the accompanying Supplement Materials for more details about the neural models.

## References

[1] Kosiak M. (1959). Etiology and pathology of ischemic ulcers. Arch. Phys. Med. Rehabil..

[2] Braden B., Bergstrom N. (1987). A conceptual schema for the study of the etiology of pressure sores. Rehabil. Nurs..

[3] Grey J.E., Harding K.G., Enoch S. (2006). Pressure ulcers. BMJ.

[4] Gefen A., Brienza D.M., Cuddigan J., Haesler E., Kottner J. (2021). Our contemporary understanding of the aetiology of pressure ulcers/pressure injuries. Int. Wound J..

[5] Blair M.J., Jones J.D., Woessner A.E., Quinn K.P. (2020). Skin Structure–Function Relationships and the Wound Healing Response to Intrinsic Aging. Adv. Wound Care.

[6] Kottner J., Black J., Call E., Gefen A., Santamaria N. (2018). Microclimate: A critical review in the context of pressure ulcer prevention. Clin. Biomech..

[7] Sun P.C., Jao S.H.E., Cheng C.K. (2005). Assessing foot temperature using infrared thermography. Foot Ankle Int..

[8] Kelechi T.J., Michel Y. (2007). A Descriptive Study of Skin Temperature, Tissue Perfusion, and Tissue Oxygen in Patients With Chronic Venous Disease. Biol. Res. Nurs..

[9] Sayre E., Kelechi T., Neal D. (2007). Sudden increase in skin temperature predicts venous ulcers: A case study. J. Vasc. Nurs. Off. Publ. Soc. Peripher. Vasc. Nurs..

[10] Stern M.D., Lappe D.L., Bowen P.D., Chimosky J.E., Holloway G., Keiser H., Bowman R. (1977). Continuous measurement of tissue blood flow by laser-Doppler spectroscopy. Am. J. Physiol.-Heart Circ. Physiol..

[11] Ratheesh K.M., Seah L.K., Murukeshan V.M. (2016). Spectral phase-based automatic calibration scheme for swept source-based optical coherence tomography systems. Phys. Med. Biol..

[12] Meleppat R.K., Miller E.B., Manna S.K., Zhang P., Pugh E.N., Zawadzki R.J. (2019). Multiscale Hessian filtering for enhancement of OCT angiography images. Proceedings of the Ophthalmic Technologies XXIX.

[13] Ioannou S., Gallese V., Merla A. (2014). Thermal infrared imaging in psychophysiology: Potentialities and limits. Psychophysiology.

[14] Ring E.F.J., Ammer K. (2012). Infrared thermal imaging in medicine. Physiol. Meas..

[15] Grayson J. (1988). Responses of the microcirculation to hot and cold environments. Pharmacol. Ther..

[16] Lipsitz L.A. (2002). Dynamics of stability: The physiologic basis of functional health and frailty. J. Gerontol. A Biol. Sci. Med. Sci..

[17] Goldberger A.L., Peng C.K., Lipsitz L.A. (2002). What is physiologic complexity and how does it change with aging and disease?. Neurobiol. Aging.

[18] Goldberger A.L., Amaral L.A.N., Hausdorff J.M., Ivanov P.C., Peng C.K., Stanley H.E. (2002). Fractal dynamics in physiology: Alterations with disease and aging. Proc. Natl. Acad. Sci. USA.

[19] Langemo D.K., Brown G. (2006). Skin fails too: Acute, chronic, and end-stage skin failure. Adv. Skin Wound Care.

[20] Meijer J.H., Schut G.L., Ribbe M.W., Goovaerts H.G., Nieuwenhuys R., Reulen J.P., Schneider H. (1989). Method for the measurement of susceptibility to decubitus ulcer formation. Med. Biol. Eng. Comput..

[21] Van Marum R.J., Meijer J.H., Ribbe M.W. (2002). The relationship between pressure ulcers and skin blood flow response after a local cold provocation. Arch. Phys. Med. Rehabil..

[22] Liao F., Yang T.D., Wu F.L., Cao C., Mohamed A., Jan Y.K. (2019). Using Multiscale Entropy to Assess the Efficacy of Local Cooling on Reactive Hyperemia in People with a Spinal Cord Injury. Entropy.

[23] Costa M., Goldberger A.L., Peng C.K. (2002). Multiscale entropy analysis of complex physiologic time series. Phys. Rev. Lett..

[24] Porta A., Bari V., Ranuzzi G., De Maria B., Baselli G. (2017). Assessing multiscale complexity of short heart rate variability series through a model-based linear approach. Chaos.

[25] Rapp M.P., Bergstrom N., Padhye N.S. (2009). Contribution of skin temperature regularity to the risk of developing pressure ulcers in nursing facility residents. Adv. Skin Wound Care.

[26] Bergstrom N., Horn S.D., Rapp M.P., Stern A., Barrett R., Watkiss M. (2013). Turning for Ulcer ReductioN: A Multisite Randomized Clinical Trial in Nursing Homes. J. Am. Geriatr. Soc..

[27] Padhye N.S., Bergstrom N., Rapp M.P., Etcher L., Redeker N.S. Pressure ulcer risk detection from complexity of activity. Proceedings of the 2017 39th Annual International Conference of the IEEE Engineering in Medicine and Biology Society (EMBC).

[28] Da Rosa Silva C.F., Santana R.F., de Oliveira B.G.R.B., do Carmo T.G. (2017). High prevalence of skin and wound care of hospitalized elderly in Brazil: A prospective observational study. BMC Res. Notes.

[29] Bauer K., Rock K., Nazzal M., Jones O., Qu W. (2016). Pressure Ulcers in the United States’ Inpatient Population From 2008 to 2012: Results of a Retrospective Nationwide Study. Ostomy Wound Manag..

[30] Demarré L., Verhaeghe S., Annemans L., Van Hecke A., Grypdonck M., Beeckman D. (2015). The cost of pressure ulcer prevention and treatment in hospitals and nursing homes in Flanders: A cost-of-illness study. Int. J. Nurs. Stud..

[31] Paulden M., Bergstrom N., Horn S., Rapp M., Stern A., Barrett R.S., Watkiss M., Krahn M. (2014). Turning for Ulcer Reduction (TURN) Study: An Economic Analysis. Ont. Health Technol. Assess. Ser..

[32] Bergstrom N., Braden B.J., Laguzza A., Holman V. (1987). The Braden Scale for Predicting Pressure Sore Risk. Nurs. Res..

[33] Valencia J.F., Porta A., Vallverdu M., Claria F., Baranowski R., Orlowska-Baranowska E., Caminal P. (2009). Refined Multiscale Entropy: Application to 24-h Holter Recordings of Heart Period Variability in Healthy and Aortic Stenosis Subjects. IEEE Trans. Biomed. Eng..

[34] Manis G., Aktaruzzaman M., Sassi R. (2017). Bubble Entropy: An Entropy Almost Free of Parameters. IEEE Trans. Biomed. Eng..

[35] Richman J.S., Moorman J.R. (2000). Physiological time-series analysis using approximate entropy and sample entropy. Am. J. Physiol. Heart Circ. Physiol..

[36] Faul F., Erdfelder E., Lang A.G., Buchner A. (2007). G*Power3: A flexible statistical power analysis program for the social, behavioral, and biomedical sciences. Behav. Res. Methods.

[37] Kring D.L. (2007). Reliability and validity of the Braden Scale for predicting pressure ulcer risk. J. Wound Ostomy Cont. Nurs..

[38] NPUAP Pressure Injury Stages|The National Pressure Ulcer Advisory Panel—NPUAP. https://npiap.com/page/PressureInjuryStages.

[39] Flood M.W., Grimm B. (2021). EntropyHub: An open-source toolkit for entropic time series analysis. PLoS ONE.

[40] Ribeiro M., Henriques T., Castro L., Souto A., Antunes L., Costa-Santos C., Teixeira A. (2021). The Entropy Universe. Entropy.

[41] Bandt C., Pompe B. (2002). Permutation entropy: A natural complexity measure for time series. Phys. Rev. Lett..

[42] Cuesta-Frau D., Miró-Martínez P., Oltra-Crespo S., Jordán-Núñez J., Vargas B., González P., Varela-Entrecanales M. (2018). Model Selection for Body Temperature Signal Classification Using Both Amplitude and Ordinality-Based Entropy Measures. Entropy.

[43] Arundo Analytics (2020). ADTK (version 0.6.2). https://github.com/arundo/adtk.

[44] Magagnin V., Bassani T., Bari V., Turiel M., Maestri R., Pinna G.D., Porta A. (2011). Non-stationarities significantly distort short-term spectral, symbolic and entropy heart rate variability indices. Physiol. Meas..

[45] MacKinnon J.G. (2010). Critical Values for Cointegration Tests.

[46] R Core Team (2022). R: A Language and Environment for Statistical Computing.

[47] RStudio Team (2022). RStudio: Integrated Development Environment for R.

[48] (2020). JMP 15 Fittting Linear Models.

[49] Tibshirani R. (1996). Regression Shrinkage and Selection Via the Lasso. J. R. Stat. Soc. Ser. B Methodol..

[50] Zou H. (2012). The Adaptive Lasso and Its Oracle Properties. J. Am. Stat. Assoc..

[51] (2020). JMP 15 Predictive and Specialized Modeling.

[52] Porta A., Bari V., Marchi A., Maria B.D., Castiglioni P., Rienzo M.d., Guzzetti S., Cividjian A., Quintin L. (2015). Limits of permutation-based entropies in assessing complexity of short heart period variability. Physiol. Meas..

[53] Stefanovska A., Bracic M., Kvernmo H. (1999). Wavelet analysis of oscillations in the peripheral blood circulation measured by laser Doppler technique. IEEE Trans. Biomed. Eng..

[54] Porta A., Bari V., Maria B.D., Cairo B., Vaini E., Malacarne M., Pagani M., Lucini D. (2018). Peripheral Resistance Baroreflex During Incremental Bicycle Ergometer Exercise: Characterization and Correlation With Cardiac Baroreflex. Front. Physiol..

[55] Pagani M., Montano N., Porta A., Malliani A., Abboud F.M., Birkett C., Somers V.K. (1997). Relationship Between Spectral Components of Cardiovascular Variabilities and Direct Measures of Muscle Sympathetic Nerve Activity in Humans. Circulation.

[56] Hodges G.J., Mallette M.M., Tew G.A., Saxton J.M., Moss J., Ruddock A.D., Klonizakis M. (2017). Effect of age on cutaneous vasomotor responses during local skin heating. Microvasc. Res..

[57] Mufti A., Maliyar K., Syed M., Pagnoux C., Alavi A. (2020). Approaches to Microthrombotic Wounds: A Review of Pathogenesis and Clinical Features. Adv. Skin Wound Care.

[58] Virtanen P., Gommers R., Oliphant T.E., Haberland M., Reddy T., Cournapeau D., Burovski E., Peterson P., Weckesser W., Bright J. (2020). SciPy 1.0: Fundamental Algorithms for Scientific Computing in Python. Nat. Methods.

[59] Kanazawa T., Kitamura A., Nakagami G., Goto T., Miyagaki T., Hayashi A., Sasaki S., Mugita Y., Iizaka S., Sanada H. (2016). Lower temperature at the wound edge detected by thermography predicts undermining development in pressure ulcers: A pilot study. Int. Wound J..

[60] Bennett S.L., Goubran R., Knoefel F. Long term monitoring of a pressure ulcer risk patient using thermal images. Proceedings of the 2017 39th Annual International Conference of the IEEE Engineering in Medicine and Biology Society (EMBC).

[61] Jiang X., Hou X., Dong N., Deng H., Wang Y., Ling X., Guo H., Zhang L., Cai F. (2020). Skin temperature and vascular attributes as early warning signs of pressure injury. J. Tissue Viability.

[62] Wang Y., Jiang X., Yu K., Shi F., Qin L., Zhou H., Cai F. (2021). Infrared Thermal Images Classification for Pressure Injury Prevention Incorporating the Convolutional Neural Networks. IEEE Access.

[63] Polak A., Kucio C., Kloth L.C., Paczula M., Hordynska E., Ickowicz T., Blaszczak E., Kucio E., Oleszczyk K., Ficek K. (2018). A Randomized, Controlled Clinical Study to Assess the Effect of Anodal and Cathodal Electrical Stimulation on Periwound Skin Blood Flow and Pressure Ulcer Size Reduction in Persons with Neurological Injuries. Ostomy Wound Manag..

