# Supplementary material for: Pressure Injury Link to Entropy of Abdominal Temperature
Source: Entropy (Basel). 2022 Aug 15;24(8):1127. doi: 10.3390/e24081127 (PMC9407490; doi:10.3390/e24081127)
Supplement: Supplementary file 1 [file entropy-24-01127-s001.zip › entropy-1803336-supplementary.pdf]

Neural

Validation: Random KFold

Model NTanH(1)NGaussian(1)

Training

prinj

Measures	Value
Generalized RSquare	0.9636653
Entropy RSquare	0.9261962
RMSE	0.0620281
Mean Abs Dev	0.0483488
Misclassification Rate	0
-LogLikelihood	1.0584261
Sum Freq	21

Confusion Matrix

Actual	Predicted Count	
prinj	Control	Injury
Control	12	0
Injury	0	9

Confusion Rates

Actual	Predicted Rate	
prinj	Control	Injury
Control	1.000	0.000
Injury	0.000	1.000

Validation

prinj

Measures	Value
Generalized RSquare	0.0341604
Entropy RSquare	0.0190146
RMSE	0.4484521
Mean Abs Dev	0.2892699
Misclassification Rate	0.2
-LogLikelihood	3.3010731
Sum Freq	5

Confusion Matrix

Actual	Predicted Count	
prinj	Control	Injury
Control	3	0
Injury	1	1

Confusion Rates

Actual	Predicted Rate	
prinj	Control	Injury
Control	1.000	0.000
Injury	0.500	0.500

Neural

Model NTanH(1)NGaussian(1)

Estimates

Parameter	Estimate
H1_1:scaling_bubben	-1.82796
H1_1:scaling_sampen	-1.21886
H1_1:Intercept	2.638025
H1_2:scaling_bubben	0.225045
H1_2:scaling_sampen	10.56485
H1_2:Intercept	-18.405
prinj(0):H1_1	-4.88405
prinj(0):H1_2	-44.0571
prinj(0):Intercept	-2.25265

Diagram

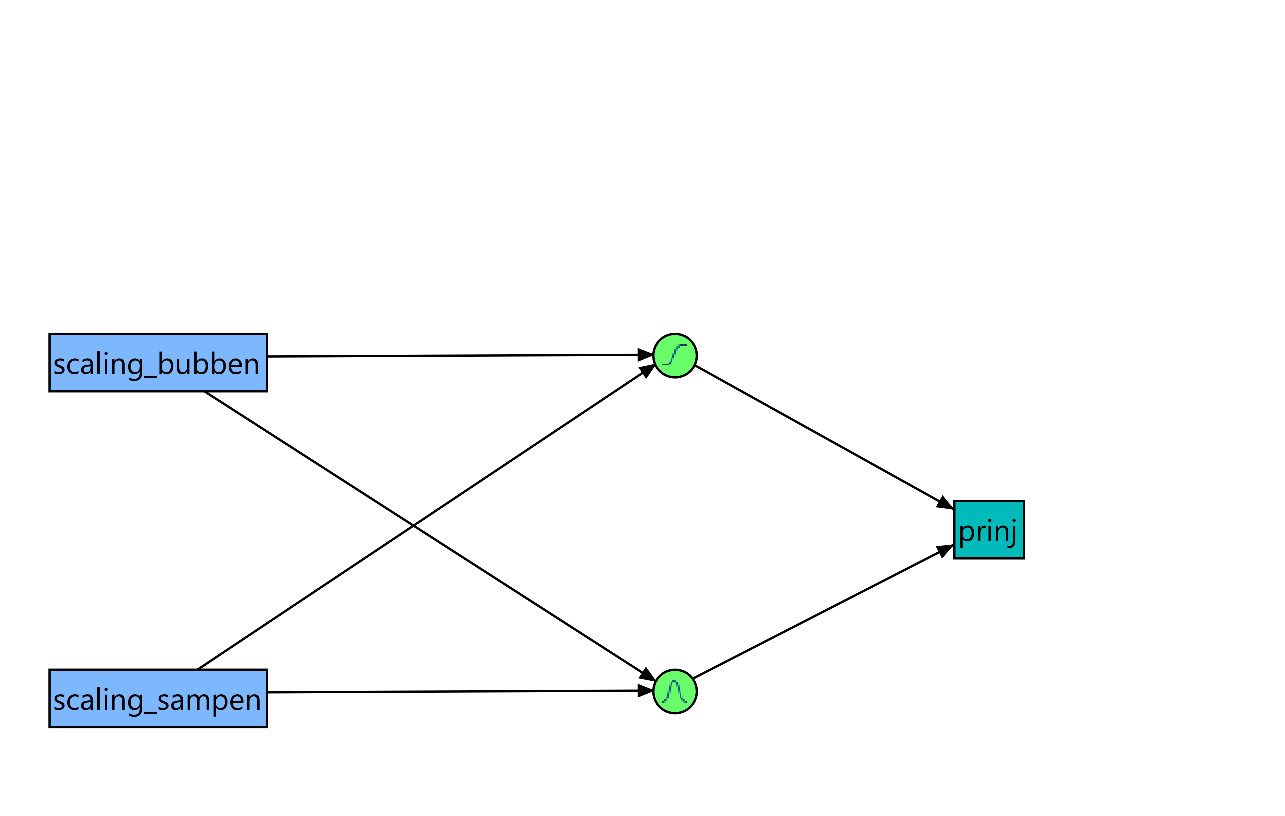

Neural

Model NTanH(1)NGaussian(1)

Training

Receiver Operating Characteristic

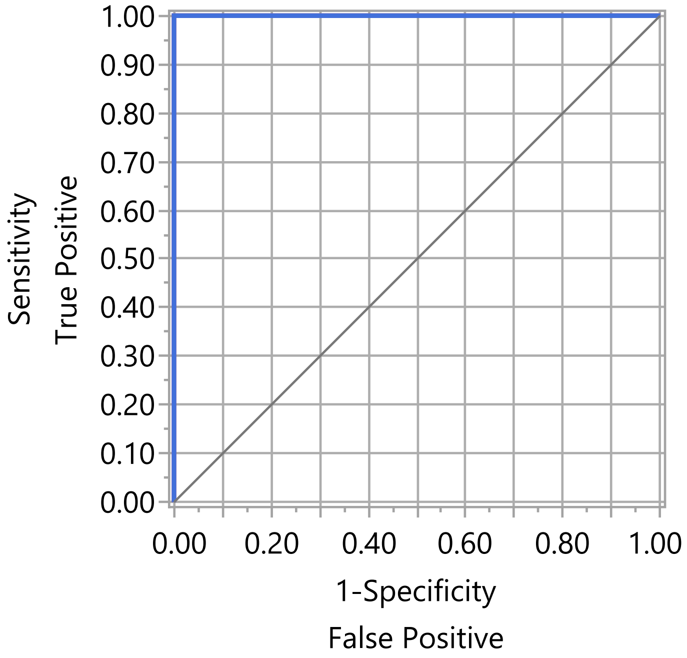

prinj	Area
Control	1.0000
Injury	1.0000

Validation

Receiver Operating Characteristic on Validation Data

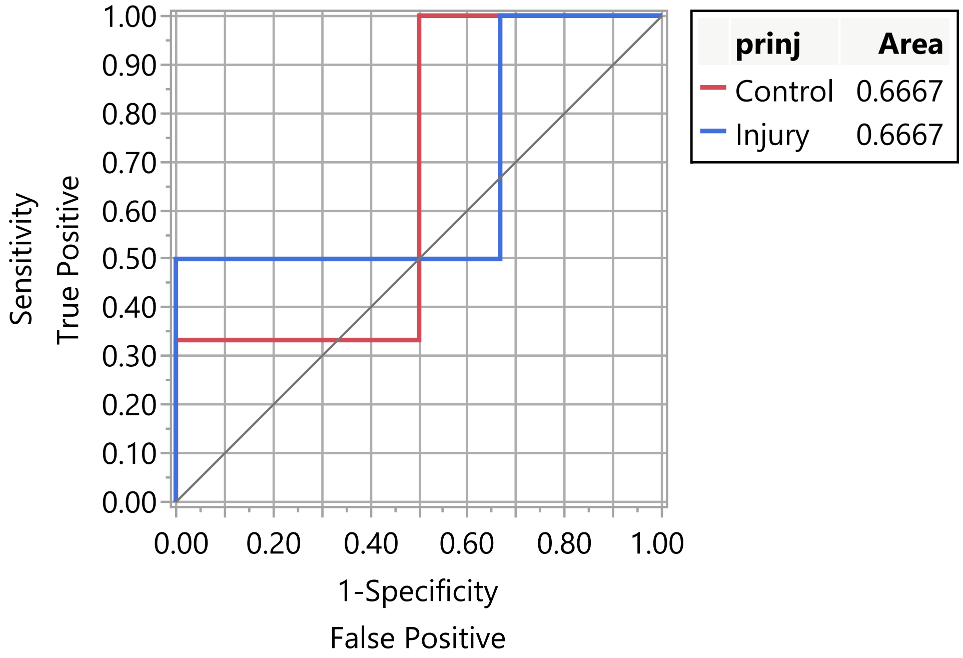

Neural

Validation: Random KFold

Model NTanH(1)NGaussian(1)

Training

prinj

Measures	Value
Generalized RSquare	0.992294
Entropy RSquare	0.9837139
RMSE	0.0333034
Mean Abs Dev	0.0105238
Misclassification Rate	0
-LogLikelihood	0.2335601
Sum Freq	21

Confusion Matrix

Actual	Predicted Count	
prinj	Control	Injury
Control	12	0
Injury	0	9

Confusion Rates

Actual	Predicted Rate	
prinj	Control	Injury
Control	1.000	0.000
Injury	0.000	1.000

Validation

prinj

Measures	Value
Generalized RSquare	0.9929095
Entropy RSquare	0.9851774
RMSE	0.0211172
Mean Abs Dev	0.0097455
Misclassification Rate	0
-LogLikelihood	0.0498788
Sum Freq	5

Confusion Matrix

Actual	Predicted Count	
prinj	Control	Injury
Control	3	0
Injury	0	2

Confusion Rates

Actual	Predicted Rate	
prinj	Control	Injury
Control	1.000	0.000
Injury	0.000	1.000

Neural

Model NTanH(1)NGaussian(1)

Estimates

Parameter	Estimate
H1_1:scaling_bubben	-0.10164
H1_1:scaling_sampen	0.73766
H1_1:bbs_tot	-0.56848
H1_1:Intercept	6.722045
H1_2:scaling_bubben	-0.06503
H1_2:scaling_sampen	1.018416
H1_2:bbs_tot	-0.38726
H1_2:Intercept	5.025514
prinj(0):H1_1	-311.103
prinj(0):H1_2	-334.964
prinj(0):Intercept	109.417

Neural

Model NTanH(1)NGaussian(1)

Diagram

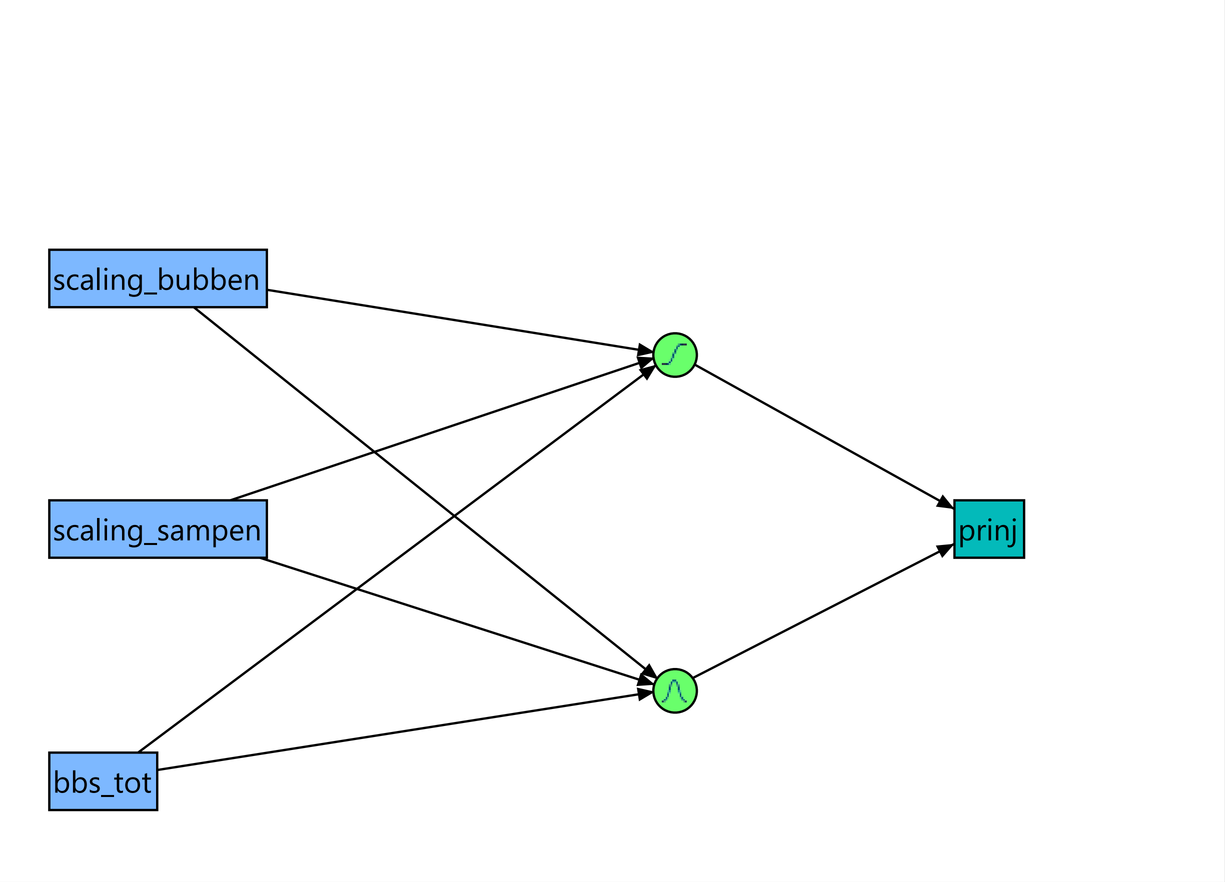

Neural

Model NTanH(1)NGaussian(1)

Training

Receiver Operating Characteristic

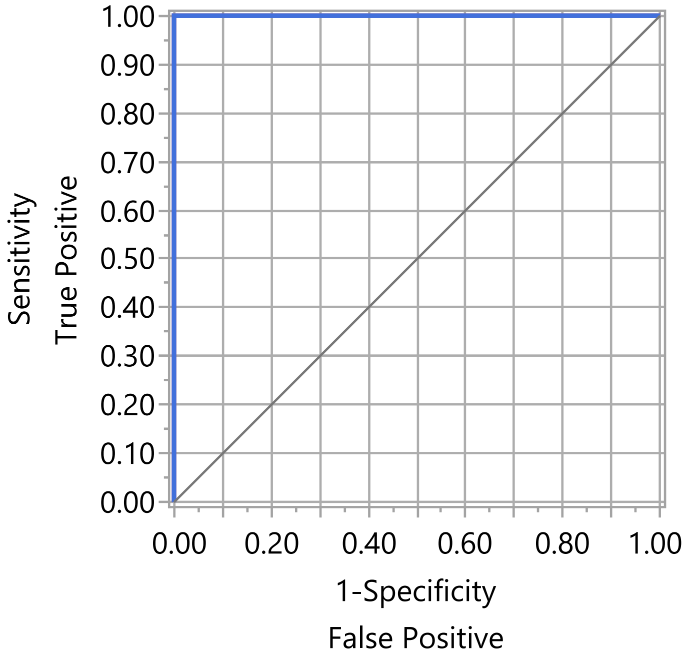

prinj	Area
Control	1.0000
Injury	1.0000

Validation

Receiver Operating Characteristic on Validation Data

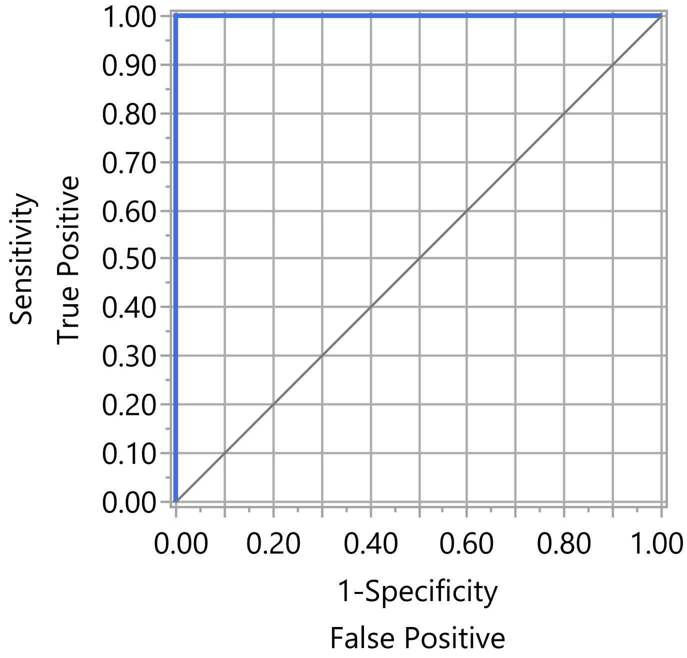

prinj	Area
Control	1.0000
Injury	1.0000
